# Cycle Development in a Mini-Freeze Dryer: Evaluation of Manometric Temperature Measurement in Small-Scale Equipment

**DOI:** 10.1208/s12249-021-02014-w

**Published:** 2021-04-26

**Authors:** Tim Wenzel, Margit Gieseler, Ahmad M. Abdul-Fattah, Henning Gieseler

**Affiliations:** 1 GILYOS GmbH, Friedrich-Bergius-Ring 15, 97076 Wuerzburg, Germany; 2 Division of Pharmaceutics, Freeze Drying Focus Group, Friedrich-Alexander University (FAU) Erlangen-Nürnberg, 91058 Erlangen, Germany; 3 CSL Behring GmbH, Emil-von-Behring-Straße 76, 35041 Marburg, Germany

**Keywords:** freeze drying, process analytical technology, cycle development, manometric temperature measurement, scale-up

## Abstract

The objective of this research was to assess the applicability of manometric temperature measurement (MTM) and SMART™ for cycle development and monitoring of critical product and process parameters in a mini-freeze dryer using a small set of seven vials. Freeze drying cycles were developed using SMART™ which automatically defines and adapts process parameters based on input data and MTM feedback information. The freeze drying behavior and product characteristics of an amorphous model system were studied at varying wall temperature control settings of the cylindrical wall surrounding the shelf in the mini-freeze dryer. Calculated product temperature profiles were similar for all different wall temperature settings during the MTM-SMART™ runs and in good agreement with the temperatures measured by thermocouples. Product resistance profiles showed uniformity in all of the runs conducted in the mini-freeze dryer, but absolute values were slightly lower compared to values determined by MTM in a LyoStar™ pilot-scale freeze dryer. The resulting cakes exhibited comparable residual moisture content and optical appearance to the products obtained in the larger freeze dryer. An increase in intra-vial heterogeneity was found for the pore morphology in the cycle with deactivated wall temperature control in the mini-freeze dryer. SMART™ cycle design and product attributes were reproducible and a minimum load of seven 10R vials was identified for more accurate MTM values. MTM-SMART™ runs suggested, that in case of the wall temperature following the product temperature of the center vial, product temperatures differ only slightly from those in the LyoStar™ freeze dryer.

## INTRODUCTION

Due to the increasing number and spectrum of (bio-)pharmaceutical and diagnostic products, freeze drying is a method of increasing importance to ensure long-term stability of these sensitive active pharmaceutical ingredients (1–4). The typically high value of biopharmaceuticals increases the need for indicative studies in small-scale freeze drying equipment to reduce the financial risk during development (5, 6). However, cycles developed in miniaturized equipment are typically difficult to scale-up owing to non-representative conditions.

Innovative process analytical technology (PAT) tools enable precise monitoring and control of the freeze drying process with the objective of a reliable and reproducible cycle performance (7). Additionally, the non-invasive determination and comparison of critical product attributes offers clear benefits in terms of process transfer between different freeze dryers and successful scale-up to larger units (8, 9). An example of a non-invasive PAT tool for real-time determination of key process and product parameters is manometric temperature measurement (MTM). The technology relies on quickly (i.e., less than a second) isolating the chamber from the condenser for 25 s (10). The resulting pressure rise curves are analyzed by non-linear regression analysis with the MTM equation which allows real-time determination of both the vapor pressure of ice at the sublimation interface (P_ice_) and the resistance of the dried product layer (R_p_) (11). Based on P_ice_ and R_p_, data for the product temperature at the sublimation interface (T_p-MTM_), the heat flow into the product vial (dQ/dt), the actual ice thickness, the temperature at the bottom of the vial (T_b-MTM_), the vial heat transfer coefficient (K_v_), and sublimation rate (dm/dt) are instantaneously calculated by the software based on basic steady-state heat and mass transfer equations (10, 11). Previous studies showed good concordance of the batch-average product temperature obtained by MTM and the temperatures measured by thermocouples (TCs) until about two-thirds of primary drying in case of amorphous formulations (11). Further benefits of MTM are real-time process monitoring, instant provision of essential product information, i.e., by the R_p_ profiles, and characterization of heat flow into the product (12–15). However, certain limits were also identified restricting the use for particular formulations or conditions, such as water readsorption tendencies of the dried layer during the MTM measurements using highly concentrated amorphous materials (13). Additionally, freeze dryer-specific features must be taken into consideration for reliable MTM values. Most importantly, a sufficiently fast isolation valve closing time (less than 1 s), the compliance with the respective minimum load (depending on the ratio between sublimation area and effective chamber volume, A_p, total_/V_eff_) and a low leak rate (below 30 mTorr/h) are crucial for a sufficiently fast pressure rise and accurate values obtained by the curve fit (10, 13).

Information gained by MTM about the critical parameters and optimal process settings during a freeze drying run can be expanded by the SMART™ feature to facilitate cycle development in one single experiment (13, 16). Based on selected input parameters, such as the critical formulation temperature and general formulation characteristics, the SMART™ freeze dryer uses feedback information from MTM to maintain T_p-MTM_ below, but close to the critical formulation temperature by balancing the appropriate amount of heat through adjustment of shelf temperature (T_s_) and chamber pressure (17). This potential to increase cost and time efficiency is particularly useful during early development, e.g., for a new formulation or container system (18, 19).

A mini-freeze dryer (LyoCapsule™, SP Scientific, Gardiner, NY, USA) was developed with the objective to enable cycle development and optimization using only seven product vials, thereby limiting the financial risk during development for high-value biologics, such as antibody-drug conjugates or proteins (20, 21). The drying chamber of the LyoCapsule™ (LC) is composed of a small, circular shelf surrounded by a cylindrical wall and a temperature-controlled radiation plate above that can be used for stoppering similar to larger scale equipment. In addition to TC ports and pirani and capacitance manometers for pressure control, the machine can be equipped with innovative PAT tools such as tunable diode laser absorption spectroscopy (TDLAS) or MTM (22). The complementary use of the mini-freeze dryer during process development, e.g., by performing indicative experiments for novel drugs, formulations, or freeze drying configurations, offers several benefits due to the low material consumption and short preparation times. For instance, more process variables can be tested in small scale to evaluate the robustness of a formulation for cycle optimization, and troubleshooting can be performed exploiting the opportunity to scale-down a process and optimize process conditions in the small unit to avoid rejection of batches (23, 24). However, heat transfer differences are expected to result in atypical drying conditions (25). To provide scalable cycles, freeze drying conditions which reflect the situation in larger systems have to be created. With the aim of achieving comparable product quality, product temperature during primary drying needs to be controlled, which requires adjustment of the heat input (26). For this reason, the temperature-controlled cylindrical wall surrounding the shelf shields the vial array from radiation effects and enables modification of heat transfer by adjusting the wall temperature (T_wall_) to comply with the conditions present in a larger freeze dryer. However, a downside of process development in a mini-freeze dryer is the limited number of product vials available for comprehensive analytical characterization. Therefore, reliable information about process performance and product quality obtained by innovative PAT tools is mandatory, particularly if the same PAT tools for monitoring and control are consistently deployed during development throughout different scales.

This study investigated the applicability of MTM in combination with SMART™ cycle design in miniaturized equipment for the first time. MTM data and SMART™ cycle design in the mini-freeze dryer are compared to a larger freeze dryer. In addition, the effect of the T_wall_ control setting on process parameters and on the reliability of MTM values is extensively studied. To optimize the MTM procedure in the mini-freeze dryer, the impact of ice sublimation area on the accuracy of product temperature measurement is explored and limits regarding the minimum load are defined. Furthermore, the reproducibility of cycle design and MTM performance is investigated and reasons for R_p_ deviations are discussed for evaluation of the reliability of this PAT tool in a small system.

## MATERIALS AND METHODS

### Materials

Ph. Eur. certified D(+)-sucrose was purchased from Sigma-Aldrich Chemie GmbH (Steinheim, Germany). Water for injection (B. Braun Melsungen AG, Melsungen, Germany) was used for the preparation of all solutions. TopLyo® serum tubing vials (20R and 10R) and standard serum tubing vials (4R) were obtained from Schott AG (Mainz, Germany). Westar® RS igloo stoppers were kindly provided by West Pharmaceuticals (Eschweiler, Germany). All chemicals were at least of analytical grade and used without further purification.

### Methods

#### Preparation of Freeze Drying Cycles

**Fig. 1:**
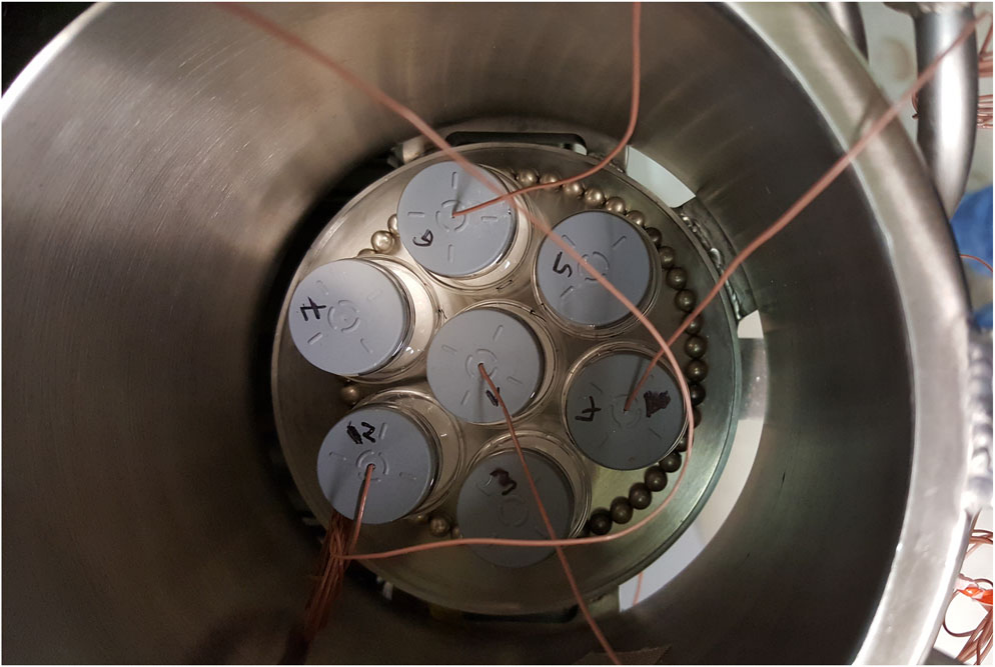
Example image of the LC mini-freeze dryer shelf with a hexagonal packaging array and magnetic beads holding the vials in place. The temperature-controlled cylindrical wall can be seen surrounding the shelf

Sucrose was dissolved in water for injection at a concentration of 50 mg/mL. The sample solutions were filtered using 0.45 μm cellulose acetate syringe filters (VWR International, Radnor, PA, USA) prior to filling of the vials. The vials were semi-stoppered and placed on the shelves in a hexagonal packing profile. In the Lyostar™ (LS) freeze dryer (SP Scientific, Gardiner, NY, USA), a stainless steel tray with a stainless steel frame was used to transfer the vials into the freeze dryer. One shelf was fully loaded with one row of empty dummy vials surrounding the product vials and acting as a thermal barrier. The bottom of the tray was removed prior to freeze drying. Additionally, aluminum foil was placed on the interior side of the front door as radiation shielding. This setup was used to reduce the edge vial effect caused by increased radiative heat transfer from the chamber door and walls (11, 27). Product temperature at the vial bottom was monitored using 36 gauge thin wire TCs (Omega Engineering, Newport, CT, USA). In the LC freeze dryer (SP Scientific, Gardiner, NY, USA), a ring of magnetic particles (*d* = 5 mm) adjusted to the respective perimeter of the seven vial array was used to keep the vials in a hexagonal packing (Fig. 1). No further radiation shields were used in the LC.

#### Applicability and Reproducibility of MTM-SMART™ Cycle Design in the LyoCapsule™

Freeze drying cycles were performed using a LC mini-freeze dryer. MTM-SMART™ cycles were conducted with a 50 mg/mL sucrose solution to evaluate the capability to control product temperature at a defined target temperature by adjusting T_s_ and chamber pressure. Three freeze drying cycles were performed to investigate the impact of T_wall_ control on SMART™ performance: T_wall_ control was disabled (LC A), T_wall_ was controlled at T_s_ (LC B), and T_wall_ was controlled at the product temperature of the center vial as measured by TC (T_b-TC, center_, LC C). T_wall_ was controlled at the respective temperatures during the entire freeze drying cycle. 10R vials (ice sublimation area A_p_ = 3.66 cm^2^) with a fill volume of 3 mL were used. The collapse temperature of the sucrose solution (−33°C) was determined in advance by light transmission freeze dry microscopy and used as input parameter for the SMART™ system (28–30). During primary drying, information obtained by MTM, such as dm/dt, R_p_, T_p-MTM_, and T_b-MTM_, is used as feedback information for the SMART™ system to optimize process conditions. MTM measurements were performed in 60-min intervals during primary drying. The temperature at the bottom of the cake was measured using three TCs placed bottom-center within the product vials (two edge vials and the center vial). The designed cycles were compared with respect to selected T_s_ setpoints, average T_b-TC_, primary drying times, and MTM process data such as T_p-MTM_ and R_p_. The accuracy of the MTM performance was assessed by comparison of T_b-MTM_ and T_b-TC_. A reference cycle was performed in a LS pilot-scale freeze dryer.

To assess the reproducibility of SMART™ cycle design (such as setpoints for T_s_ and chamber pressure) and obtained product parameters (particularly the product temperature and R_p_ profiles), triplicate SMART™ freeze drying experiments with T_wall_ following T_b-TC, center_ were performed for the 50 mg/mL sucrose solution in 10R vials.

#### Limitations of MTM Measurements in the LyoCapsule™

By changing vial size and number, the impact of a decreasing ice sublimation area on the MTM data analysis was analyzed. Experiments in the LC were performed with different vial numbers (7 20R; 7, 5, 3 and 1 10R; 37 4R vials). This corresponded to total ice sublimation areas of 40.2, 25.6, 18.3, 11.0, 3.7, and 51.8 cm^2^, respectively. Pressure rise curves as well as the deviations between T_b-MTM_ and T_b-TC_ were compared for the LC cycles and a LS reference cycle with a load of 198 10R vials.

#### Characterization of Freeze Dried Samples

##### Scanning Electron Microscopy

Inner product morphology of the lyophilisates was assessed using scanning electron microscopy (SEM). Two product cakes for each batch were extracted intact from the vial by cutting the vial about 5 mm above the cake. The samples were split in half along the cylinder axis and sputtered twice with gold at 7 mA for 5 to 10 min each (Hummer Sputter System, Anatech Ltd., Union City, CA, USA). A Tescan Vega\\xmu Scanning Electron Microscope (Brno, Czech Republic) with an acceleration voltage of 20 kV was used to analyze the samples. SEM analysis was performed on vials without TCs. Average pore sizes along the central axis were determined by measuring ten pores near the top, middle, and bottom of the products, respectively (31).

##### Residual Moisture Measurement

Karl Fischer coulometric titration was used to determine residual water content. Two vials for each LC experiment and six vials for each LS experiment (three edge vials and three center vials, respectively) were used for analysis. Only non-TC vials were used for the residual moisture analysis. Between 40 and 80 mg of sample aliquots were filled into glass vials under dry atmosphere (glove box, 20–25°C, relative humidity < 1%) and crimped. The exact sample weight was recorded by weighing the empty and filled vials using a Mettler Toledo (AT261 DeltaRange®) analytical balance. Sample vials were placed in an 874 Karl Fischer Oven Sample Processor (Metrohm, Filderstadt, Germany) at a temperature of 80°C. The oven temperature was evaluated to be sufficient for complete water extraction of the processed freeze dried products in this study. Higher oven temperatures did not result in an increase in the extracted water content during the analysis. The water was transferred into a Karl Fischer Moisture Analyzer 831 KF (Metrohm, Filderstadt, Germany) by purging the vial headspace with dry nitrogen at 60 mL/min, and moisture content was recorded in percent. The precision of the measurement was verified using a crystalline water standard with 1% water content (Hydranal™ Water Standard KF Oven).

#### Statistical Tests

Statistical differences between the means of the values for the nucleation temperatures and residual moisture obtained in different freeze dryers and in the LC using different T_wall_ settings, respectively, were analyzed using a 1-way ANOVA test. Differences were considered statistically significant for *p* ≤ 0.05.

## RESULTS AND DISCUSSION

### MTM-SMART™ Cycle Design Using an Amorphous Excipient Solution (50 mg/mL Sucrose)

**Fig. 2:**
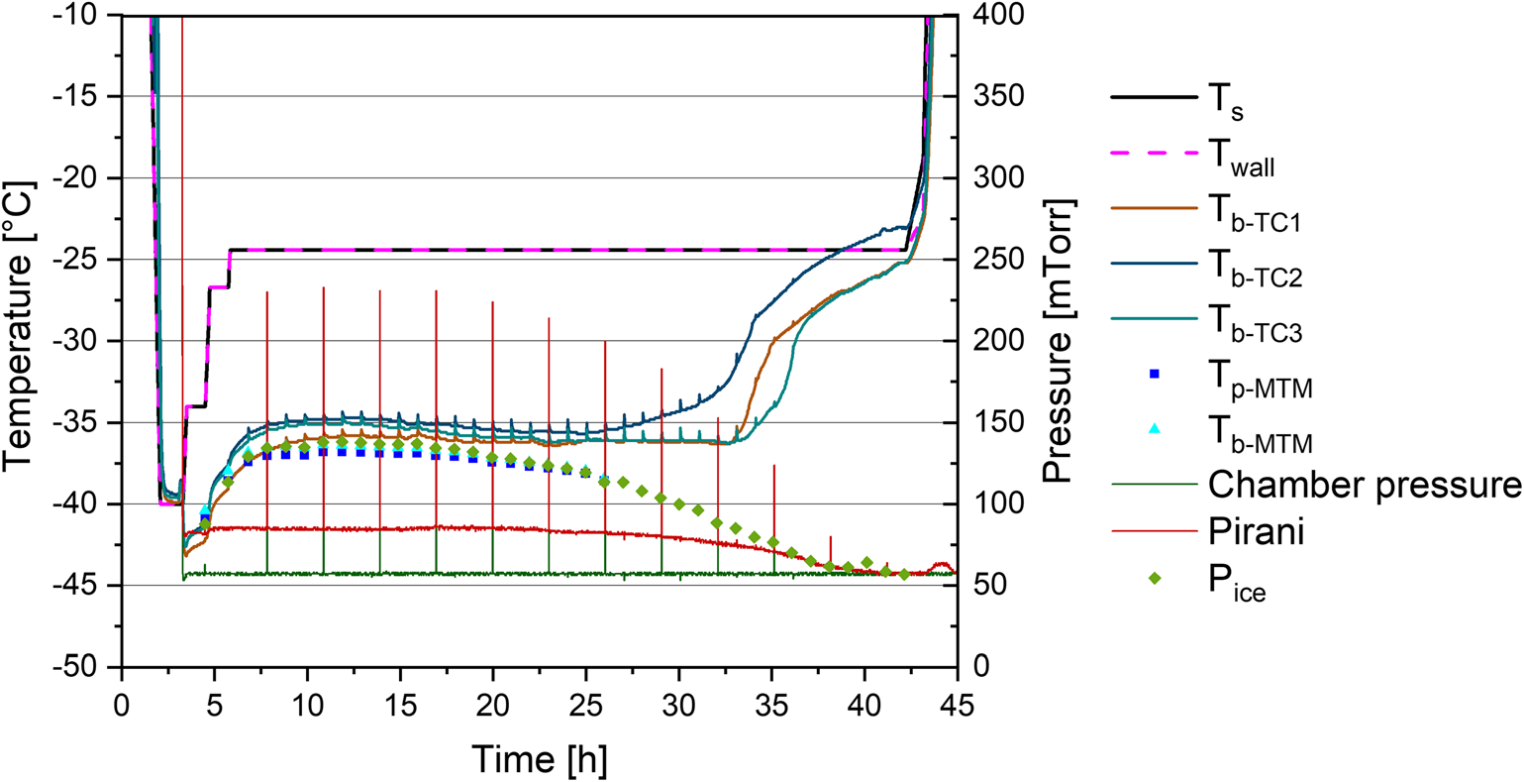
Cycle design by MTM-SMART™ for a 50 mg/mL sucrose solution in the LC mini-freeze dryer with T_wall_ following T_s_ (LC B)

A detailed plot of the LC B freeze drying cycle with T_wall_ following T_s_ is shown in Fig. 2. It should be noted that system data was exported in 1-min intervals. Because of the 25 s duration of the drying chamber isolation during the MTM procedure and the fast return to the chamber pressure setpoint afterwards, a pressure increase is not visible in the exported data for every MTM data point. Furthermore, the setpoints for T_s_ and chamber pressure were chosen and adapted by the system to maintain the product temperature below the collapse temperature (−33°C) throughout the process. Because of the T_wall_ control setting, the T_s_ setpoints selected by the SMART™ system had a direct impact on T_wall_. As shown in Fig. 2, T_s_ was adjusted twice at the beginning of primary drying until reaching a final setpoint of −24.4°C during steady-state conditions and this temperature was maintained until the end of primary drying. T_b-MTM_ values were consistently within 1°C of T_b-TC_ during the first half of primary drying. A trend towards lower T_b-MTM_ values compared to T_b-TC_ can be observed which is in agreement with the MTM bias towards the coldest vials of the batch described in the literature (11). After this period, the inaccuracy of the MTM measurements increases based on drying heterogeneity owing to a decrease of effective ice sublimation area (11). Due to the safety margin of 3°C the SMART™ software used, T_b-TC_ values remained at least 1.5°C below the collapse temperature under applied conditions confirming the capability of MTM-SMART™ to select appropriate process conditions for this excipient solution. The endpoint of primary drying as determined by comparative pressure measurement was consistent with the decrease in P_ice_ to chamber pressure obtained by MTM measurements. Thus, the MTM method was sensitive enough in the mini-freeze dryer to determine the primary drying endpoint and automatically select the start of secondary drying for an efficient cycle.

### Reproducibility of Cycle Design and Process Parameters

The identical setting and equal input information for the software should engender comparable primary drying conditions and, in turn, provide a consistent product temperature profile and primary drying time in the triplicate reproducibility experiments. As the most challenging configuration affecting reproducibility of SMART™ constitutes the conditions in LC C (T_wall_ = T_b-TC, center_), this setup was chosen to test the reproducibility of MTM-SMART™. Potential differences in placement of the TC within the center vial may have a substantial impact on the temperature reading and, in turn, on the T_wall_ setting. Consequently, the heat input into the vials could be modified resulting in deviating product temperatures. The setpoints for chamber pressure and T_s_, as well as the cycle adjustments during the first hours of primary drying selected by the SMART™ freeze dryer for the triplicate runs, showed good reproducibility (Table I). T_s_ setpoints deviated by less than 2°C between the three runs, and in none of the cycles the initial chamber pressure setpoint of 57 mTorr was changed. Differences in primary drying times were negligible (approximately 6%) between the three experiments. The comparable process conditions are expected to result in similar product temperature over time profiles. The average product temperatures deviated by less than 0.5°C between the three runs which is most critical for a reproducible product quality (32). It was maintained below the collapse temperature (−33°C) in all cycles. R_p_ profiles were consistent throughout the three runs and only negligible deviations of other MTM process data that were attributed to regular inter-batch heterogeneity were found.

**Table I:** Process Conditions and Product Parameters of Triplicate MTM-SMART™ Cycles During Steady-State Primary Drying for 3 mL 50 mg/mL Sucrose in 10R Vials in the LC Mini-Freeze Dryer. T_wall_ = T_b-TC, center_ in All Cycles

	Run 1	Run 2	Run 3
Number of vials	7	7	7
Nucleation temperature [°C]^a^	− 12.9 ± 1.0	− 14.0 ± 1.5	− 13.8 ± 2.0
Primary drying time [h]	35.0	35.1	33.0
T_s_ (set) [°C]	−20.4	−20.9	−19.5
T_b-TC_ [°C]	−35.4 ± 0.5	−34.6 ± 0.5	−34.5 ± 0.4
T_p-MTM_ [°C]	−36.8 ± 0.1	−36.7 ± 0.1	−36.7 ± 0.2
T_b-MTM_ [°C]	−36.3 ± 0.1	−36.3 ± 0.1	−36.2 ± 0.2
P_ice_ [mTorr]	138.05 ± 1.31	139.36 ± 1.48	139.05 ± 3.21
dQ/dt [cal/h/vial]	56.25 ± 1.81	53.69 ± 1.57	59.07 ± 2.12
R_p_ [cm^2^ * Torr * h/g]	3.15 ± 0.12	3.33 ± 0.12	3.02 ± 0.16
dm/dt [g/h/vial]	0.094 ± 0.005	0.090 ± 0.000	0.100 ± 0.000

^a^1-way ANOVA: *F* = 0.7277, *p* = 0.5124

### SMART™ Cycle Design and Process Data as a Function of Different Wall Temperature Control Settings

#### Comparability of Temperature Profiles and Primary Drying Times

**Fig. 3:**
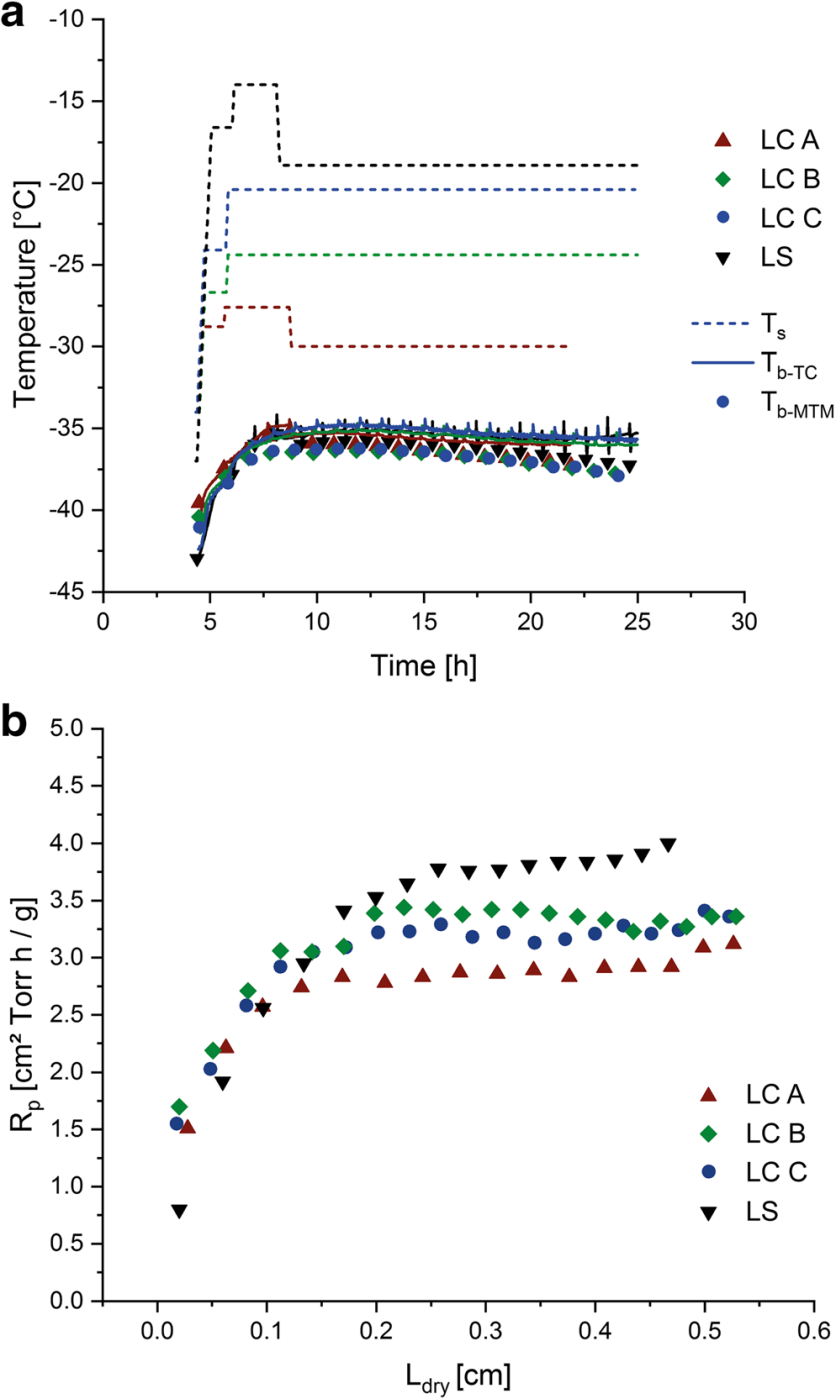
**a** T_s_ (dashed lines) and T_b_ (T_b-MTM_: symbols; T_b-TC_: solid lines) and **b** R_p_ profiles from the MTM-SMART™ freeze drying cycles with different T_wall_ control settings in the LC (LC A: T_wall_ control disabled; LC B: T_wall_ = T_s_; LC C: T_wall_ = T_b-TC, center_) and the LS reference cycle

Overlays of the T_b_ profiles including applied T_s_ in the MTM-SMART™ runs using different T_wall_ settings are shown in Fig. 3a and Table II. Owing to the strong impact of T_wall_ on the heat input into the product vials, T_s_ setpoints deviated strongly between the three cycles. Whereas the initial chamber pressure setpoint of 57 mTorr was maintained unchanged, the T_s_ setpoints differed by almost 10°C between the different runs using disparate T_wall_ control settings. The pronounced reduction of T_s_ in LC A with disabled T_wall_ control highlights the need for restraining the heat input from the shelves in order to avoid the product temperature exceeding the collapse temperature. Product temperature heterogeneity within the batch was a concern for the accuracy of the MTM measurements because of the reported bias of MTM data towards the coldest vials in a batch (11). However, despite the temperature differential of approximately 35°C between T_wall_ and T_s_ in LC A, a satisfactory homogeneity of product temperatures within the batch was found with less than 1°C difference between T_b-TC, center_ and the surrounding edge vials. A possible explanation for this small difference could be a thermal shielding effect of the magnetic beads holding the vials in the LC in the hexagonal packaging array (Fig. 1), similar to the effect of the metal guardrail in the LS (11). This observation diminished concerns of higher inaccuracy of the MTM values due to drying heterogeneity between edge and center vials. The T_s_ and T_wall_ differential between the different T_wall_ settings had no impact on product temperature profiles. A very good agreement of product temperatures for all conditions in the mini-freeze dryer were observed and comparability to the profiles in a larger unit was confirmed (Fig. 3a). Depending on T_wall_, the cycles took around 34–39 h to complete and the endpoints of primary drying as measured by the decrease in P_ice_ to chamber pressure (MTM) were in close agreement with the comparative pressure measurement for all settings.

**Table II:** Comparison of Product and Process Parameters During Steady-State Primary Drying of MTM-SMART™ Runs With 3 mL 50 mg/mL Sucrose Solution in 10R Vials Using Different T_wall_ Settings in the LC in Comparison to a Pilot-Scale LS Freeze Dryer

	LS	LC A T_wall_ control disabled	LC B T_wall_ = T_s_	LC C T_wall_ = T_b-TC, center_
Number of vials	198	7	7	7
Nucleation temperature [°C]^a^	− 11.1 ± 3.1	− 13.0 ± 1.7	− 13.3 ± 2.3	− 12.9 ± 1.0
Primary drying time [h]	37.7	33.8	38.9	35.0
T_s_ (set) [°C]	−18.9	−30.0	−24.4	−20.4
T_wall_ [°C]	-	5.30	−24.40	−34.04
T_b-TC_ [°C]	−35.3 ± 0.5	−35.3 ± 0.4	−35.1 ± 0.6	−35.4 ± 0.5
T_p-MTM_ [°C]	−36.2 ± 0.1	−36.6 ± 0.1	−36.9 ± 0.1	−36.8 ± 0.1
T_b-MTM_ [°C]	−35.8 ± 0.2	−36.1 ± 0.0	−36.5 ± 0.1	−36.3 ± 0.1
P_ice_ [mTorr]	146.80 ± 2.28	141.00 ± 2.00	136.59 ± 1.49	138.05 ± 1.31
dQ/dt [cal/h/vial]	55.94 ± 0.57	63.26 ± 1.50	55.13 ± 2.60	56.25 ± 1.81
R_p_ [cm^2^ * Torr * h/g]	3.58 ± 0.29	2.85 ± 0.04	3.28 ± 0.18	3.15 ± 0.12
dm/dt [g/h/vial]	0.094 ± 0.011	0.109 ± 0.004	0.094 ± 0.005	0.094 ± 0.005

^a^1-way ANOVA: *F* = 0.9596, *p* = 0.4390

*LS*, LyoStar™; *LC*, LyoCapsule™

While the data in this study showed reasonably comparable product temperature profiles and primary drying times regardless of T_wall_ control, T_wall_ should not be reduced too far below the product temperatures. The temperature bias of MTM measurements towards cold vials is hypothesized to be caused by readsorption of water vapor from “hot vials” on “cold” vials (14). Similarly, water readsorption on the cylindrical wall surrounding the shelf and therefore an increased error in T_b-MTM_ may be expected when T_wall_ in the mini-freeze dryer is reduced to values too far below the product temperature of the vials.

Whereas T_wall_ control had a pronounced impact on process and MTM data in the LC, the impact of the wall temperature in the LS is considered negligible for this investigation. Wall temperatures during sublimation in a LS freeze dryer at T_s_ = −10°C and 100 mTorr chamber pressure were measured at −10°C for the side walls, between −5 and 5°C for the back wall and between 15 and 20°C for the chamber door (33). The reader is advised that the side walls are not the chamber walls but the walls of the shelf stack in the LS. Consequently, most vials in the LS are only exposed to the side walls which have temperatures comparable to the shelf surface. Furthermore, the combination of more pronounced thermal shielding in the LS and the bias of MTM data towards the coldest or center vials of the batch further diminishes the influence of wall temperature on MTM performance in the LS (14).

#### Comparability of Product Resistance, Vapor Pressure of Ice, and Sublimation Rates

For all T_wall_ control settings, the resulting cakes exhibited a low R_p_ to water vapor flow that increased non-linearly with dry layer thickness (L_dry_) as measured by MTM (Fig. 3b). Despite similar T_b-TC_ profiles at any given wall temperature setting, different R_p_ values were observed. In addition, the profiles obtained in the LC were consistently lower compared to the R_p_ profile in the LS. For example, at L_dry_ = 0.3 cm, the R_p_ was 2.86 cm^2^ × Torr × h/g in LC A, 3.42 cm^2^ × Torr × h/g in LC B, 3.22 cm^2^ × Torr × h/g in LC C, and 3.77 cm^2^ × Torr × h/g in the LS. Thus, R_p_ was 1.3 times lower in LC A, 1.1 times lower in LC B, and 1.2 times lower in LC C compared to the reference value measured in the LS.

The mismatch in R_p_ profiles — particularly between the LC A cycle with deactivated T_wall_ control and the LS — cannot be explained by a disparity in the P_ice_, as these profiles showed good consistency and only slightly deviated from the P_ice_ profiles of the settings with reduced T_wall_ in the LC. Furthermore, nucleation temperatures were not significantly different in all experiments regardless of T_wall_ control in the LC and freeze dryer (Table II) and consequently can be ruled out as a relevant factor for the observed differences as well. A possible explanation for the different R_p_ profiles could be inaccuracy of MTM data because of the relatively slow pressure rise due to the low sublimation area in relation to chamber volume for all T_wall_ control settings. The influence of the rate of pressure increase on MTM data is further discussed in a later section of this investigation in context with the reduced load experiments. An elevated dm/dt was observed in case of LC A. This could have been caused by increased heat transfer into the vials because of the relatively large temperature differential between T_wall_ and T_s_ of approximately 35°C in this experiment. This observation is in line with the slightly reduced primary drying time and lower average R_p_ compared to the LC B and C cycles and the cycle in the LS (34). Additionally, small differences in pore morphology as discussed in the next section may also have contributed to the lower R_p_ values for the LC A cycle.

### Characterization of Freeze Dried Products: Impact of the Wall Temperature Control on Product Quality Attributes

**Fig. 4:**
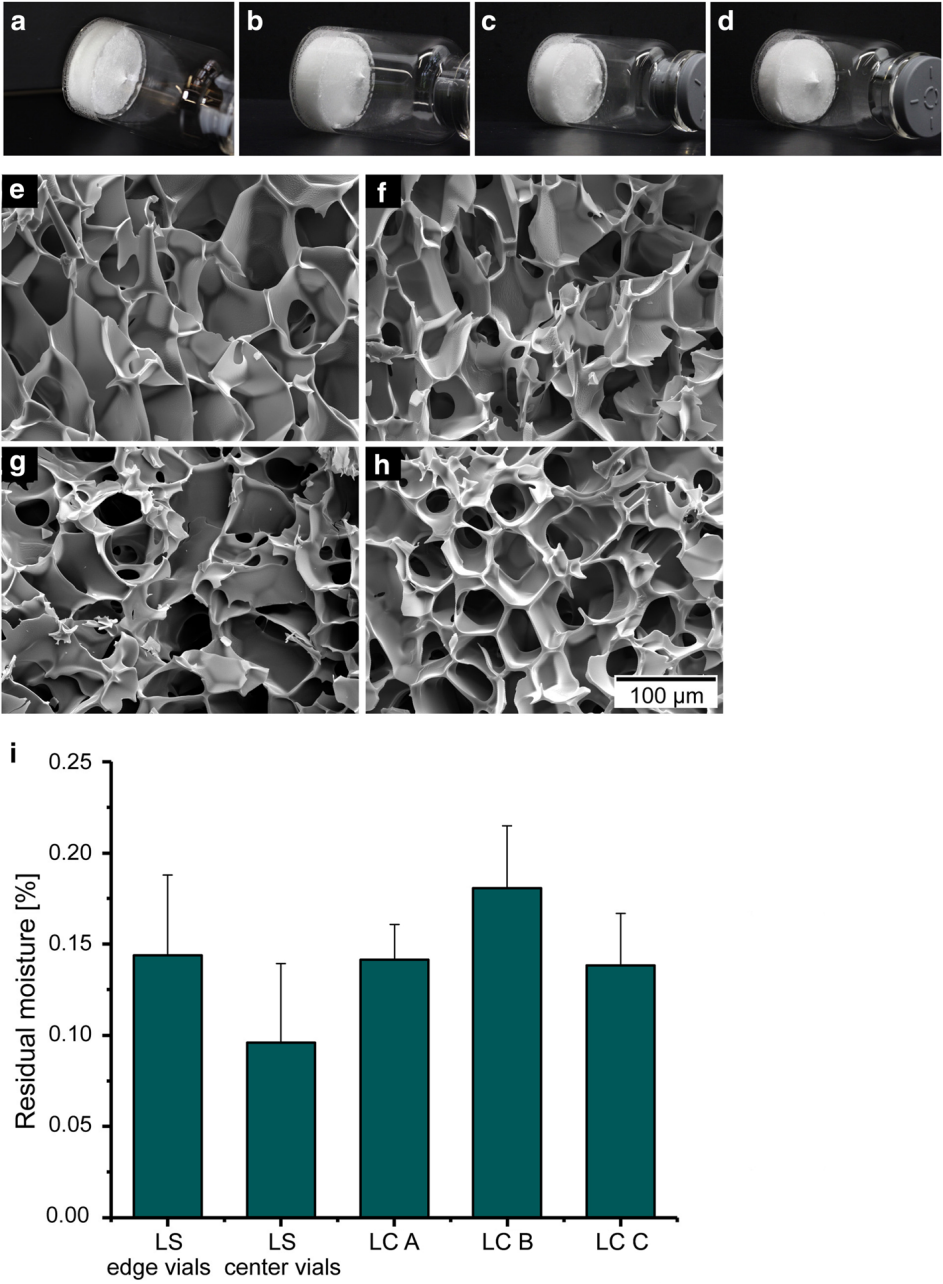
**a**–**d** Product pictures and **e**–**h** example SEM images of the middle section of products freeze dried from 10R vials for 50 mg/mL sucrose using different T_wall_ settings in the LC compared to reference products in the LS. **a**, **e** LC A, T_wall_ control disabled, **b**, **f** LC B, T_wall_ = T_s_, **c**, **g** LC C, T_wall_ = T_b-TC, center_, **d**, **h** LS reference products. **i** Average residual moisture content of 50 mg/mL sucrose samples processed under these different conditions

As depicted in Fig. 4a–d, a certain level of shrinkage was observed for all products of the cycles using different freeze dryers and T_wall_ settings. This is expected behavior for sucrose solutions during freeze drying, regardless of the T_s_ setpoint during primary drying (35, 36). The extent of shrinkage was comparable in all products freeze dried with different conditions. Overall cake appearance was elegant and uniform across all batches (37, 38).

Example SEM images for the different T_wall_ control batches and the LS reference cycle are shown in Fig. 4e–h. Average pore sizes along the central axis near the top, middle, and bottom are listed in Table III. SEM analysis was particularly important for the detection of intra-vial heterogeneity. This was expected because of the varying temperature differentials between the top and bottom of the vial during freezing depending on the investigated T_wall_ control settings. Product defects were not observed in the freeze dried samples. Pore sizes appeared slightly larger in the upper section of the cake in the samples processed in cycle LC A. A potential explanation for this observation could be differences in radiative heat transfer during freezing: the higher T_wall_ in case of LC A led to slightly higher product temperatures during freezing which could explain the larger pore sizes in the upper section of the cake. No differences in the intra-vial pore size distribution could be observed for LC B and LC C products with activated T_wall_ control or in the LS.

**Table III:** Average Pore Sizes in the Center of the 50 mg/mL Sucrose Products Obtained in the LC With Different T_wall_ Settings in Comparison to a Pilot-Scale LS Freeze Dryer

		LS	LC A T_wall_ control disabled	LC B T_wall_ = T_s_	LC C T_wall_ = T_b-TC, center_
Average pore sizes [μm]	Top	94.4 ± 11.8	120.5 ± 13.9	88.4 ± 15.3	82.5 ± 16.1
	Middle	105.0 ± 10.8	97.0 ± 14.5	98.0 ± 11.6	91.8 ± 10.2
	Bottom	84.0 ± 8.3	80.0 ± 8.9	81.5 ± 10.4	79.6 ± 16.5

*LS*, LyoStar™; *LC*, LyoCapsule™

The similar pore structure observed in all samples was reflected by equivalent residual moisture contents suggesting comparable desorption rates during secondary drying. All samples of the different cycles were low in moisture content with less than 0.2% average residual moisture measured for all products (Fig. 4i). No significant differences between the freeze dryers and T_wall_ control settings were observed (1-way ANOVA, *F* = 2.3726, *p* = 0.1106).

In summary, the similar product temperature profiles of all cycles in the mini-freeze dryer using active T_wall_ control (LC B and C) resulted in a comparable cake appearance, pore morphology, and residual moisture content. These product quality attributes were also comparable to the products processed in a larger freeze dryer. It should be highlighted that these observations were made with a relatively simple amorphous model system. Further investigations with more complex formulations are necessary to elucidate if this conclusion is also applicable to other systems.

### Limitations of the MTM Technique for Use in Small-Scale Equipment

**Fig. 5:**
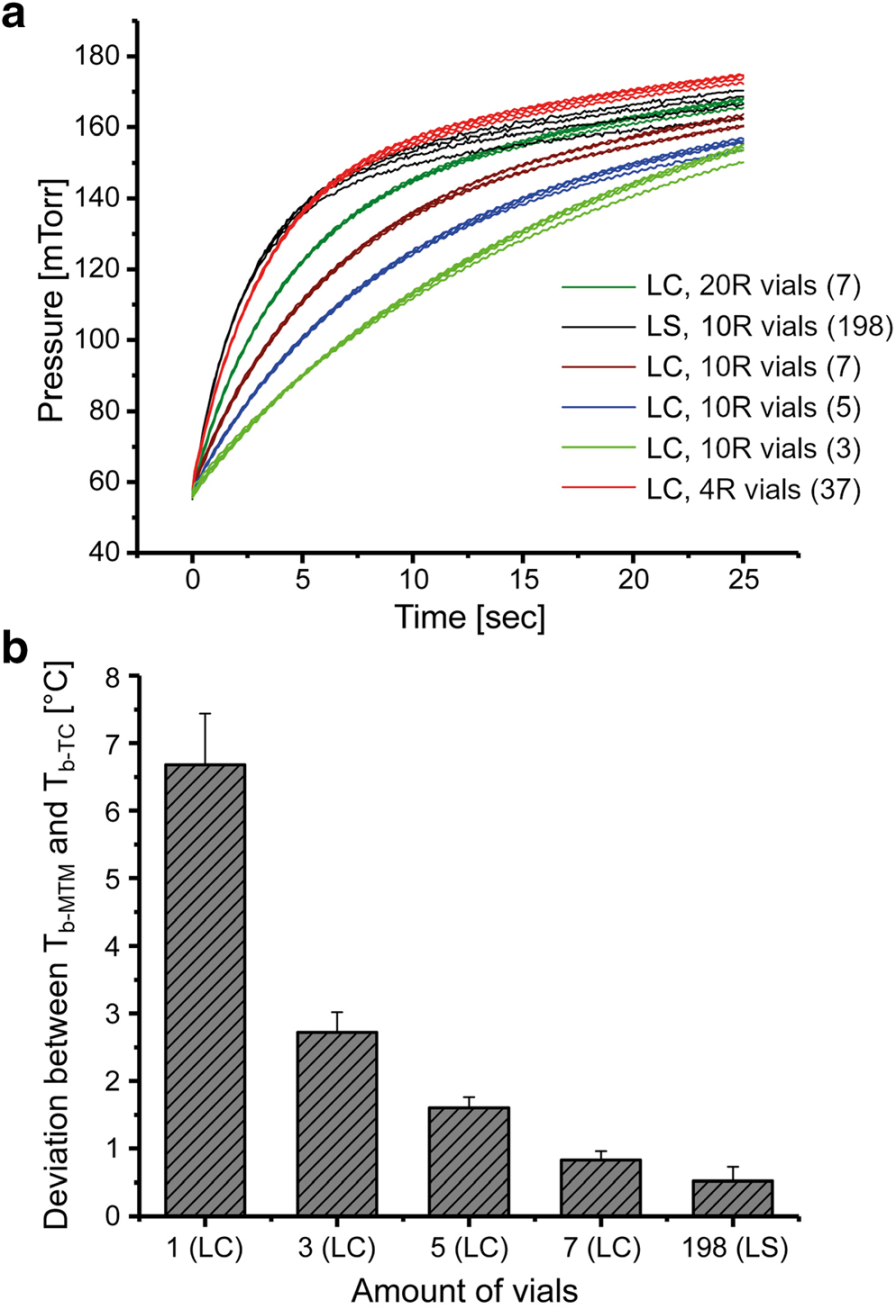
**a** Experimental pressure rise curves for 50 mg/mL sucrose using different loading conditions and freeze dryers. The curves represent the pressure increase under steady-state primary drying conditions recorded in 0.1-s intervals. **b** Deviation between average T_b-TC_ and T_b-MTM_ during steady-state primary drying under different loading conditions in the LC and LS freeze dryers

Figure 5a shows the effect of the ice sublimation area (i.e., the number of vials) on the shape of the pressure rise curves using a 50 mg/mL sucrose solution and identical drying conditions in the LC. As clearly visible, the pressure rise curves obtained in the mini-freeze dryer showed a reduced slope compared to the reference curves in the larger freeze dryer. Particularly in case of reducing the load from seven to five or three 10R vials, respectively, the exponential portion was too long for a sufficient product temperature-dominated plateau phase to develop within the measurement time. In contrast, performance of a freeze drying cycle using 37 4R vials (corresponding to an ice sublimation area of 51.8 cm^2^ compared to 40.2 cm^2^ using seven 20R vials and 25.6 cm^2^ using seven 10R vials, respectively) resulted in a fast rise very similar to the curve progression in the pilot-scale freeze dryer.

Accurate determination of critical product and process parameters by MTM requires a sufficiently rapid chamber pressure rise during the MTM collection period (11). A too slow pressure rise may lead to a long resistance-dominated exponential pressure rise phase relative to the second, temperature-dominated phase. Consequently, the accuracy of the calculated product parameters, such as T_b-MTM_, may be impaired. For freeze drying runs in a laboratory-scale freeze dryer (FTS Dura-Stop) with a chamber volume of 52 L, the sublimation area needs to be at least 150 cm^2^ corresponding to approximately 50 5R vials (11). The beta version of the LC comprises a cylindrical drying chamber with an effective chamber volume (V_eff_) of 10.6 L determined by the pressure rise method (13). The ratio of the total ice sublimation area of all product vials to the effective chamber volume of the freeze dryer (A_p, total_/V_eff_) defines the required minimum product load for a reliable prediction of the product temperature by MTM (11, 13).

The effect on the product temperature determination by MTM is illustrated in Fig. 5b. T_b-MTM_ and T_b-TC_ were in good agreement for seven vials in the LC and a full tray of product vials in the LS. The error in the calculation of T_b-MTM_ increases with a reduction of the ice sublimation area (e.g., a “full load” of seven 10R vials resulted in a small temperature deviation of less than 1°C, but with decreasing load and A_p, total_/V_eff_ ratio, the T_b_ deviation increases to approx. 1.6°C for five vials, to approx. 2.7°C for three vials, and to more than 6°C for one single 10R vial). It should be noted that the accuracy of the T_b-MTM_ and R_p_ determination depends on the slope of the pressure rise curves (11). Even under full load conditions using seven vials, a relatively slow chamber pressure rise during the valve closure period is found for this model system. This could have led to a small systematic error in MTM data and may explain the discrepancies observed in the R_p_ profiles between the LC cycles and the LS reference cycle discussed earlier. It is reasonable to assume that the error in MTM data will increase with formulations with higher R_p_ and therefore less pronounced slopes of the pressure rise curves. The validity of MTM data should consequently even be investigated under full load conditions for formulations with higher R_p_. However, endpoint detection by a decrease in P_ice_ was in good agreement with the endpoint by comparative pressure measurement even using a very small load of three vials suggesting a high sensitivity of measured P_ice_ values.

The experimental results obtained support the conclusion that a minimum load of seven vials — corresponding to “full load conditions” in this small system — is imperative for accurate MTM product temperature determination in the LC, and, in turn, reasonable SMART™ cycle development. Thus, the minimum ratio A_p, total_/V_eff_ is calculated to be 2.4 for the LC, which is close to the ratio of 2.7 found for the laboratory-scale and pilot-scale freeze dryers of different sizes (Dura-Stop and LS) (11, 13).

Additionally, several requirements to the design and characteristics of the freeze dryer are relevant prerequisites for the use of MTM. For example, a leak rate below 30 mTorr/h is necessary for accurate calculations (13). In terms of the pressure rise curves, this is reflected by a sufficiently long plateau phase or change in slope after the initial pressure increase. If the leak rate is too high, the pressure will keep increasing too rapidly during the MTM collection period and no clear product temperature-dominated plateau phase is formed. Consequently, the non-linear regression analysis may lead to inaccurate data (11, 13). During the experiments performed in this study, the typical leak rate in the LC was determined to be within 25–33 mTorr/h (0.09–0.11 mTorr × L/s) and thus at the upper limit for acceptable MTM performance. In contrast, leak rates measured in the LS freeze dryer were in the range of 7–10 mTorr/h (0.33–0.47 mTorr × L/s). While equipment specifications are sometimes given with the volume normalized leak rates, the absolute leak rates are important for MTM performance (13). The low chamber volume of the LC freeze dryer poses a challenge to this because the lower volume normalized leak rate translates into a more pronounced absolute pressure increase compared to larger equipment.

## SUMMARY AND CONCLUSION

The present study evaluated the applicability of MTM-SMART™ for cycle development with different wall temperature control settings in the LyoCapsule™ mini-freeze dryer and compared process data and product quality attributes to a pilot-scale freeze dryer. A reasonable SMART™ cycle development was demonstrated for various wall temperature settings, and good agreement of the vapor pressure of ice with comparative pressure measurement for endpoint detection was found. Additionally, calculated T_b-MTM_ profiles showed a good conformity with the temperatures measured by temperature probes under different conditions, and reproducibility of the process and product parameters was confirmed. Control of the wall temperature at values equivalent to the product temperature of vials during primary drying led to comparable process data and product quality attributes of a 50 mg/mL sucrose system in both scales. Considering the minimum load requirement of seven 10R vials and a regular testing of the leak rate, the mini-freeze dryer with MTM-SMART™ can be used irrespective of the wall temperature settings for substantial time and resource savings during early development and optimum data comparability during scale-up to larger equipment.

## Abbreviations

A_p_: Ice sublimation area
dm/dt: Sublimation rate
dQ/dt: Heat flow into the product vial
K_v_: Vial heat transfer coefficient
LC: LyoCapsule™
L_dry_: Dry layer thickness
LS: LyoStar™
MTM: Manometric temperature measurement
PAT: Process analytical technology
P_ice_: Vapor pressure of ice at sublimation interface
R_p_: Dried product layer resistance
SEM: Scanning electron microscopy
T_b-MTM_: Product temperature at vial bottom by MTM
T_b-TC_: Product temperature at the vial bottom by TC
TC: Thermocouple
TDLAS: Tunable diode laser absorption spectroscopy
T_p-MTM_: Product temperature at sublimation interface by MTM
T_s_: Shelf temperature
T_wall_: Wall temperature in the LyoCapsule™
V_eff_: Effective chamber volume
